# 

**Published:** 1912-11-02

**Authors:** 


					A GENEROUS FRIEND.
Mr. Oswald Stoll has very kindly placed the Leicester
Palace Theatre at the disposal of the Royal Infirmary
Matinee Committee. Mr. Stoll also arranged for the free
services of the staff and orchestra, and the artistes ancf
himself bore the coet of printing in order that the whole
takings should be handed over. At the matinee on Wed-
nesday, last week, the Mayor of Leicester remarked that
the sum to be handed over would be a record?something
like ?130.
Answers to Correspondents.
We are asked by a woman, aged thirty-nine, who lives
at home and keeps house for her brother, and does in
addition outside daily work?two hours each day?for
which she receives 4s. 6d. weekly, if she must become
insured. If so, she desires to know the rates of contribu-
tion payable by herself and her employer respectively.
She also asks if the brother is counted as an employer
and is liable to pay.
As the outside work is regular, she must bo insured. The
remuneration of 4s. 6d. a week is equivalent to 9d. a day,
reckoning six days to the week, and as only two hours
are worked per day, the rate of remuneration is higher
than 2s. a day. Thus the full contribution of 6d. a week
is payable, and the employer is entitled to deduct 3d. a
week from the wage. As there does not appear to be a
contract of employment between the brother and sister,
there is no ground to support a claim for insurance in
respect of the household duties.
We are also asked the rate of contribution in the case of
a girl over twenty-one years of age who does daily work,
for which she receives ?24 yearly, with lunch and tea.
This is a case to which"we cannot reply categorically. The
lunch and tea must be taken into account in reckoning the
rate of remuneration, and it will depend on the value
placed thereon whether the full contribution is payable
or not. The employer may, if he so desire, pay the full
contribution of 6d. a week, making the deduction of 3d.
from the girl's wage, in which case she has the right of
appealing to the Insurance Commissioners should she feel
aggrieved.
Appointments.
Dr. H. Neville Crowe has been appointed hon. sur-
geon to the Worcester Ophthalmic Hospital in place of
Dr. Seymour, who has resigned.
The Colonial Secretary, it is understood, has appointed
Mr. H. J. Read, C.M.G., to represent the Colonial Office
on the committee of the School of Tropical Medicine.
The School is arranging to send an expedition (the 29th)
to Jamaica and the West Indies with the co-operation of
the Colonial Office.
The following appointments have been made at the
'Hampstead General and North-West London Hospital :?
Gynaecologist?Mr. Bryden Glendining, M.B., M.S.,
F.R.C.S. Surgeon to Out-Patients?Mr. Chad Woodward,
M.B., B.Ch., F.R.C.S. Resident Casualty Officer?Mr. B,
Sangster Simmonds, M.B., B.S., M.R.C.S., L.R.C.P.
Assistant Casualty Officer?Mr. Ronald G. Gordon, M.B.,
Ch.B., B.Sc.
Buildings Contemplated and in Progress.
Ayr.?Plans: and an estimate of, ?2,'700 have been
adopted by the Ayr Town Council for a diphtheria block
at Heathfield Hospital.
Bangor.?A contract for ?17,589 for a new workhouse
infirmary lias been placed by the Bangor and Beaumaris
Guardians.
Birmingham.?It is proposed to extend the ward accom-
modation at the Birmingham and Midland Women's Hos-
pital at a cost of about ?6,000.
Bristol.?The Guardians, with a view to future exten-
sion of their premises, have at an outlay of ?2,500 pur-
chased property adjoining St. Peter's Hospital.
Campbeltown.?Report says that additions are projected
at Campbeltown Infectious Hospital, and that Miss
Greenlees, of Burnbank, has given ?1,000 to help with
the project.
Colchester.?The Essex County Council has received
sanction to a loan of ?14,295 towards the cost of a new
asylum.
Earlestown.?Mr. Arthur Bowes, the surveyor to the
Newton-in-Makerfield Urban Council, is responsible for
the design of the new twenty-bed isolation hospital which
has just been completed at Earlestown.
Easington.?The Easington Rural District Council has
adopted by a majority of seven votes the design by Mr.
Hugh Hedley, of Sunderland, for a proposed new isolation
hospital, estimated to cost ?12,000.
Easington*.?The Rural District Council has decided to
apply for sanction to a loan of ?13,000 for erecting and
equipping a hospital.
Eccleshall.?The Guardians are applying for sanction
to a loan for an operating theatre at the workhouse.
Edinburgh.?Mr. R. M. Cameron, of 53 Great King
Street, is architect for the proposed children's home in
Crewe Street for the Parish Council. Tenders for the
work are being considered.
Exeter.?The Exeter Board of Guardians has resolved
to borrow ?7,000 for the erection of a children's home.
Gloucester.?The Gloucester Guardians have accepted
provisionally tenders of ?24,457 and ?1,553 respectively
for a new workhouse infirmary and fencing and road-
making in connection therewith. The highest of seven
tenders for the main buildings amounts to ?32,280.
Hampton.?Plans for an hospital at Hampton-on-
Thames, which Mr. T. Foster Knowles is erecting and
partly endowing, has been approved by the Urban District
Council.
Huddersfield.?Messrs. Kirk and Sons, of Hudders-
field, are architects for a new receiving home for children in
Ramsdeir Street.
Hull.?The new hospital extension at the Hessle Road
Workhouse is so far advanced that the foundation-stone
has been laid; the extension is to cost about ?16,000.
Ipswich.?It is proposed to enlarge the Guardians' In-
firmary at a cost of about ?5,500, and Mr. J. A. Scheuer-
mann, architect, has been instructed to prepare plans for
the approval of the Local Government Board.
Keighley.?The Local Government Board is asked, to
sanction a loan of ?3,000 for a cubicle isolation pavilion
by the Keighley and Bingley Joint Hospital Board.
Kilmarnock.?The infirmary is to be remodelled at a
cost of about ?12,000. Dr. Mackintosh, of Glasgow
Western Infirmary, and Mr. John Burnet, LL.D.,
A.R.S.A., are advising the directorate.
Lincoln.?The Corporation propose to embark on a
tuberculosis scheme to cost about ?10,000.
Mardy.?The Welsh National Memorial Fund for
Tuberculosis has arranged that the new block at Merthyr
Isolation Hospital shall be maintained for the treatment of
consumptives.
Middlesbrough.?The Corporation Sanitary Committee
has ordered the preparation of a scheme for a new sana-
torium.
Misterton.?The Misterton Rural District Council pro-
pose to erect an infectious hospital at Walkeringham.
Moose Jaw, Sask.?A new city hospital is contemplated
to cost ?60,000.
Neath.?The Neath Guardians are taking up a ?24,000
loan for a new graded infirmary at Penrhiewtyn.
New Westminster, B.C.?Messrs. Atkinson and Dill,
of Vancouver, have secured the contract for a hospital
at New Westminster to cost about ?27,000.
Newcastle.?The New South Wales Public Works Com-
mittee is considering a proposal to erect a hospital to cost
?46,558 at Newcastle, N.S.W.
Newton Abbot.?Mr. J. C. Beare, of Newton Abbot, is
architect for extensions at the hospital, including a
children's ward. Particulars for tender may be had on
deposit of 21s.
NOTICE TO OUR READERS.
The Editor welcomes for Institutional Housekeeping ouny con-
tribution from those engaged in the work of the commissariat i?
institutions. All accepted contributions are paid for. Queries,
answered on any subject relating to the supply, cost, storage*
distribution, or preparation of food.
138 THE HOSPITAL November 2, 1912.
BUREAU OF INFORMATION.
Rules for Correspondents.
1. Every letter, on whatever subject, must be accompanied'by
the coupon to be out from the baok cover (in6ide page) of The
Hospital, ourrent issue, and must contain the name and address
of the correspondent with pseudonym for publication if desired.
2. Replies by post cannot be given, save under exceptional
circumstances at the Editor's discretion.
3. Letters from, Approved Homes in reply to special needs pub-
lished in the Bureau should state terms and full particulars, and
toe sent prepaid under cover to the Editor of the Bureau with
coupon and name enclosed for identification.
4. Proprietors of Homes which have not yet been entered on the
List of Approved Homes, but have spare accommodation likely to
suit special needs, are invited to write for an application form for
registration. The fee for registration, which includes two an-
nouncements of the Home in the Bureau, is 5s.
5. Special needs of every description relating to those who are
sick or in difficulties are made known in these columns free of
charge.
6. All communications to be addressed to The Editor of Th*
Hospital, 28 Southampton Street, Strand, London, W.C., and
marked " Bureau of Information."
Approved Homes.
Note.?ATo Home is added to the List without direct
medical and other testimony to its good management.
Westcliff-on-Sea.? St. Ursula's, King's Road. Prin-
cipal : Miss Haslock. Slight mental and nerve cases, de-
pression, melancholia, etc. Organised system of employ-
ment for patients. Large airy premises, with sea view.
Wide and successful experience.
Worthing.?13 St. George's Road. Principal : Miss
Jane Green. Non-infectious, convalescent, elderly and
chronic invalids, not bedridden. Very moderate terms if
large room is shared. Pleasant house, with sea view and
iome comforts.
Nursing Homes Wanted.
For Tubercular Spinal Case.?Open-air treatment in
or near Bournemouth required for patient suffering from
tubercular spinal complaint. Terms from about ?3 3s.
Replies to Sister Margaret (129a).
Needs and Helpers.
Home for Case of Arthritis. ? A female patient,
mite helpless, who requires constant attention, suffering
with rheumatoid arthritis, is in need of a home where she
could be received on payment of 10s. a week. Neighbour-
hood of London preferred. If she is under forty write
to St. Cyprian's Home, 31 The Grove, Hammersmith.
.Write also to the Sister-in-Charge, St. Elizabeth's Home,
59 Mortimer Street, London, W.; and to the Alexandra
Home, 4 Francis Grove, St. George's Road, Wimbledon.
?Scotia (130x).
Convalescent Home for Lady.? The lady in Avhom
you are interested, who is recovering from very serious
illness and is straitened in means, could be received at
the Merchant Taylors' Company's Convalescent Home for
Ladies at Bognor. Apply for form to the Clerk, The
Hall, Threadneedle Street, London, E.C. It is quite free,
with free railway pass from London, and is beautifully
managed in the interests of poor gentlewomen.?Mrs. T.
Tolroy.
Miscellaneous.
Water-taps for Operating-room. ? We note you
desire information as to the best kind of wash-basin to
get for a small operating-room in a- nursing home, with
the best method of turning on the water-taps, and that
expense is an object. A basin which hasi proved satisfac-
tory after prolonged use is one fitted with a pedal-action
combination valve. This valve is worked by an arm,
which is easily and conveniently turned in a semi-circle
by the foot. It allows hot, cold, or mixed water to fall
into the basin from an overhanging delivery pipe, which
is raised above the basin to allow ample space for wash-
ing the hands in running water. The valve, etc., costs
about ?4, and the basin and delivery pipes rather under.
Perhaps the whole would cost ?7 or so. Messrs. Dent
and Hellyer, of 35 Red Lion Square, London, W.C.,
supply and fit these valves very satisfactorily to any type
of basin.?Beta.
Training and Employment.
Opening for Medical Women in Bengal.?A fully
equipped Mission Hospital for Women is in urgent need
of a woman doctor; twenty-two beds and crowded dis-
pensaries. Splendid opportunity for work. Many
patients must be left untended unless help is speedily
forthcoming. Prepaid letters, addressed in separate en-
velope to " Interested One," will be forwarded to the
right quarter.
Disposal of Nursing Home.?We know your home
well, having visited it some years ago. Should you decide
to give it into other hands, first have it valued?that is,
the actual furniture and plant?by a competent person,
and then advertise it or place it in the hands of a good
agent, with a view to meeting with a nurse who would
carry it on in the same spirit. As, however, it does not
appear that you are in a hurry to sever your connection
with it, we strongly advise you to wait awhile, in the
hope that the Bill now before Parliament which relates
to the class of sufferers you receive will become law. In
that case a great impulse will be given to the excellent
work you have been doing for so many years, and the
county authorities would probably be very glad to take
over your home, should you desire it. In either case,
valuation is the first step.?Sister-in-Charge.
Massage by Correspondence.?You could learn the
necessary physiology and anatomy through the medium
of correspondence classes, but you could not get employ-
ment unless you had been personally instructed in the
movements and had had some months at least of practice.
Moreover, the practice should be gained in an institution
for the sick.?M. T. D..
Nursing in the Empire and Abroad.
Nursing Home Wanted at Wiesbaden.?We are
advised by one of the leading physicians that this town
offers a good prospect to a nurse able to open a nursing
home for patients of moderate means. About 4,000
British subjects pass through Wiesbaden every year, of
whom quite a large proportion are invalids. The mild
climate suits many Scotch and Irish people. Hotels and
pensions cannot offer suitable accommodation to such
patients, who often suffer great inconvenience and discom-
fort for want of proper attention. A thorough knowledge
of German life, with fluency in conversation, would be
essential to success. We shall be happy to place any
correspondents who desire further particulars into com-
munication with a correspondent on the spot.
Letter-Box.
Seeker.?Glad to know you have found the home you
wanted through the medium of the Bureau.
M. Mannering (Lowestoft).?Very pleased to hear of
your new arrangement offering so good an opportunity for
winter patient at very reasonable terms.
St. Andrew's Hospital, Dollis Hill Lane, Cricklewood,
N.W.?Will the secretary of this scheme now in contem-
plation send his name and address and particulars of this
hospital to the Editor, The Hospital, 28 and 29 South-
ampton Street, Strand, London, W.C.
A New Experience for West Ham
School Children.
A very original function took place at th? West
Ham Hospital on Saturday last. For many years the
Board School teachers, now Council teachers, have en-
couraged the children attending the Council schools of
West Ham to collect for and take an interest in the
hospital, particularly the children's ward. They have a,
fund which is called the " West Ham Hospital School
Children's Fund," which fund provides an annual donation
of ?300, and finds linen and dressing-jackets for the
children's ward. It also gives special grants and keeps th?
Children's Samaritan Fund provided with money for send-
ing convalescent children away to the seaside. It also
has, at frequent intervals, given special grants in aid of
the hospital, and last year entirely renovated the interior
of the children's ward, tiling the walls, putting a new
floor, and painting and flattening the ceiling and walls
above the tiles. In order that the children might know
more intimately the work of the hospital, Mr. T. A.
Cook, the chairman, invited the teachers to arrange for
representative boys and girls from the West Ham Council
schools to visit the hospital.
On Saturday last, therefore, forty boys, representing
a large number of schools, attended the hospital at
three o'clock, and were shown through th? wards by th?
Matron and Mr. Cook, accompanied by Mr. W. Smith,
the chairman of the fund, Mr. Hildred, and Mr. E. W.
Child, hon. secretary of th? fund (schoolmasters). Th?
boys displayed great interest in the work of the hospital,
and many cam? provided with coins, which they dropped
into the boxes as opportunity occurred on their way round.
They were entertained to tea. Only two had ever been,
inside a hospital before.
On Saturday, November 16, a representative body of
girls from the Council schools will be similarly entertained,
and it is hoped to make this function an annual one.
Prizes have been offered for the best account of the visit
to the hospital written by the boys.
142 THE HOSPITAL November 2, 1912.
Gifts and Bequests.
Miss Elizabeth Anne Green, of Leicester, has left
?200 to the Female Asylum, Leicester.
The Chelsea Hospital for Women has received ?52 10s.
towards its rebuilding from the Fishmongers' Company.
The Royal Dental Hospital of London, Leicester Square,
has received ?51 10s. from the City Corporation, and ?50
?from the executors of Mr. John Yicary.
Mrs. Jane Slazenger, of Prince's Gate, has bequeathed
?1.000 each to the Crippled Boys' Homes, Wright's Lane,
Kensington, and the Aberdeen Infirmary.
Sir Ernest Hatch, the Treasurer of University College
Hospital, has received a contribution of 100 guineas from
a visitor as a mark of appreciation of the care and atten-
tion accorded to the patients in the hospital.
Mr. William Jacks, of Bristol, wholesale draper and
warehouseman, has left ?400 each to the Bristol Royal
Infirmary and the Bristol General Hospital; and ?50 each
to the Bristol Eye Hospital and the Bristol Eye Dispen-
sary.
Mrs. Agnes Black, of Coldstream and Lanton, widow
of a farmer, left public bequests amounting to nearly
?14,000, in addition to a portion of the residue of her
estate. They include ?1,000 to the Coldstream Cottage
Hospital.
Miss Mary Houldsworth, of Ayr, whose estate is
valued at ?105,695, made the following public bequests :?
Ayr County Hospital, ?3,000; London Hospital, ?5,000;
Sick Children's Hospital, Glasgow, ?5,000; and the
hospital in Glasgow to be founded for the relief of suffer-
ing on homoeopathic principles, ?5,000.
Major Samuel Nicholson, of Hove, has left ?5,000 to
the Royal Alexandra Hospital for Sick Children, Brighton,
for the endowment of a " Nicholson Ward," and after one
or two other bequests the residue of his estate npon trust
for his two sisters for life, with remainder to the Royal
Alexandra Hospital for Sick Children, Brighton.
The Secretary-Superintendent of Addenbrooke's Hospital
has recently received ?4 13s. from Mr. E. Atkinson,
head-master of S waff ham Prior School, as a contribution
to the funds of the hospital. It represents sums of one
penny per week collected by the school children from the
residents in the village, and is an instance of how even
children can help the hospital.
Obituary.
By the somewhat sudden death of Dr. Frank Montague
Pope the Leicester Royal Infirmary loses an honorary
consulting physician of high repute and one who placed
the;best years of his life at the disposal of that institu-
tion. Dr. Po_pe took his B.A. at Cambridge in 1877,
graduated M.B. London in 1881, and M.D. in 1901, and was
elected a Fellow of the Royal College of Physicians of
London in 1902. He formerly held appointments at
the Royal National Hospital for Consumption, Queen
Victoria's Sanatorium, the Great Northern Hospital, and
the Royal Hospital for Diseases of the Chest, City Road,
London. In the later years of his life he took a great
interest in the British Medical Association, of which body
he was a member of the Council and chairman of the
Science Committee. In addition to the other honours
which fell to him, he was appointed a member of the
Advisory Committee of the Society for the Feeble-
minded, "was chief surgeon to the local branch of the
St. John Ambulance Brigade, and a Lieutenant-Colonel of
the R.A.M.C. Territorial Force, 5th Northern General
Hospital. It "was only on April 27 last that Dr. Pope
resigned the senior physiciancy of the Leicester Royal
Infirmary.

				

